# Strain-controlled critical temperature in REBa_2_Cu_3_O_y_-coated conductors

**DOI:** 10.1038/srep11156

**Published:** 2015-06-11

**Authors:** Satoshi Awaji, Takumi Suzuki, Hidetoshi Oguro, Kazuo Watanabe, Kaname Matsumoto

**Affiliations:** 1 High Field Laboratory for Superconducting Materials, Institute for Materials Research, Tohoku University; 2 Kyusyu Institute for Technology

## Abstract

Recently, we succeeded in detwinning REBa_2_Cu_3_O_7_ (RE123, RE = rare-earth elements)-coated conductors by annealing under an external uniaxial strain. Using the untwinned RE123 tapes, the uniaxial-strain dependencies of the critical temperature *T*_c_ along the *a* and *b* crystal axes were investigated over a wide strain region from compression to tension. We found that the strain dependencies of *T*_c_ for the *a* and *b* axes obey a power law but exhibit opposite slopes. In particular, the maximum value of *T*_c_ is obtained when the CuO_2_ plane becomes a square, and its lattice constant is close to 0.385 nm. It is suggested that a tetragonal structure with *a* ≈ 0.385 nm is the optimum condition for a high critical temperature in high-*T*_c_ cuprates.

The external pressure is an important parameter for controlling superconductivity. In particular, the lattice distortion under an applied pressure/stress is generally related to a mechanism of superconductivity. In fact, the world’s highest critical temperature *T*_c_ of approximately 164 K was obtained under an external hydrostatic pressure of 31 GPa in HgBa_2_Ca_2_Cu_3_O_y_ (Hg1223)^1^. Recently, the onset and zero-resistivity critical temperatures were confirmed to be 159.3 K and 153.0 K, respectively, under a hydrostatic pressure of 15 GPa by another group^2^. Because the critical temperatures of most high-*T*_c_ superconducting (HTS) materials exhibit a strong external-pressure dependence, it is suggested that the lattice distortion induced by the external pressure is important to realize a high critical temperature. In addition, anisotropic pressure effects are also important because of the stacked-layer structures of HTS materials. For the REBa_2_Cu_3_O_7_ (RE123) superconductor, the pressure *P* sensitivities of *T*_c_ are much different among the crystal axes. The d*T*_c_/d*P* values are −2.0, +1.9, and −0.3 K/MPa for the *a*-, *b*-, and *c*-axes, respectively^3^. Surprisingly, the signs of the d*T*_c_/d*P* values for the *a*- and *b*-axes are opposite to each other. MacManaus-Doriscoll *et al.* pointed out the importance of the lattice strain when increasing *T*_c_ in high-*T*_c_ cuprates^4^. Although detailed experiments on the superconductivity over a wide range of uniaxial pressures are strongly required, the pressure scale of the uniaxial-pressure experiments is limited and much smaller than that of hydrostatic-pressure experiments because of the brittle nature of the superconducting material.

On the other hand, the external uniaxial-stress/strain effect of a practical superconducting wire is very important from an application viewpoint. In particular, it is well known that the large hoop stress induced by the electromagnetic force is one of the critical issues for high-field-magnet applications. The stress/strain effects on the critical current density *J*_c_ for RE123-coated conductors have been investigated^5^,^6^,^7^,^8^. In these cases, uniaxial strains up to 1% are typically applied. The 1% uniaxial strain corresponds to a uniaxial pressure of approximately 1.63 GPa, as estimated from the applied strain using an elastic constant of approximately 163 GPa^9^. This value is more than six times larger than the reported uniaxial pressure of 0.25 GPa for a single crystal^3^. Therefore, a wide range of uniaxial-pressure effects on the superconductivity can be investigated if RE123-coated conductors are used. In particular, it is very important that we control the strains from compression to tension for RE123 coated conductors using a special spring^5^,^10^. However, a serious experimental issue related to the use of RE123 coated conductors is the mixture of *a* and *b* axis domains due to the existence of twin boundaries. Because of the coexistence of different crystal-axis domains, it is considered that the strain dependency of *J*_c_ of RE123-coated conductors is very complicated^5^. Recently, we have succeeded in completely detwinning RE123-coated conductors using annealing under an applied strain^11^. In this study, we report the wide range of the uniaxial-strain dependence of *T*_c_ using detwinned coated conductors and discuss the role of the lattice distortion on *T*_c_ in the RE123 system.

## Detwinning practical RE123 tapes

For RE123 single crystals, it is easy to remove the twin boundaries by a heat treatment under an external uniaxial pressure^12^. Therefore, detwinning is routinely performed for Y123 single crystals. Twin-free Y123 single crystals have been used by many researchers for vortex physics. For RE123-coated conductors and films, however, detwinning is not easy because of the existence of a substrate and buffer layer^13^. Recently, we found that the twin boundaries in RE123 tapes can be removed by annealing under a tensile strain (strain annealing)^11^. In this technique, after the sample temperature was increased to 300 °C, a tensile strain was gradually applied up to approximately 1.0%. The applied strain was maintained as the sample was cooled to 200 °C and then released. The detailed detwinning process was reported elsewhere^11^. The samples used in this study were commercial RE123 tapes (SF4100) without artificial pinning centres produced by SuperPower Inc.^14^. The RE123-coated conductor has a multi-layered structure, and the substrate is a randomly oriented 100-μm-thick Hastelloy. MgO with a *c-*axis orientation and in-plane texture was formed on the Hastelloy by an ion-beam-assisted deposition (IBAD) technique as the textured buffer layer. RE123 was deposited onto the textured buffer layer by a chemical vapour deposition process. A 2-μm-thick Ag film was then deposited as a cap layer by a sputtering method onto the RE123 layer. The total thickness of the RE123-coated conductor is approximately 100 μm. Because the RE123-coated conductor is fabricated on a metallic substrate, and its thickness is only 0.1 mm, it is flexible and robust. Hence, we can apply a large strain to RE123.

### Internal strain evaluation under an external strain by X-ray diffraction

In order to confirm the texture and internal strain of the RE123 layers, we performed transmission X-ray diffraction in the longitudinal and transverse directions of the tapes using a Mo X-ray tube. Because Mo-Kα X-rays can penetrate deeply because of their short wavelength, we can perform a non-destructive analysis of the RE123 layer in the 40-μm-thick Cu sheath and 50-μm Hastelloy substrate. In addition, a tensile load frame is combined with the X-ray diffraction apparatus. In order to check the intrinsic strain under an external strain (macroscopic strain), we performed X-ray diffraction experiments under external applied strains as well. The detailed internal-strain measurements have been published elsewhere^15^.

### Transport measurements under strain

**Figure 1:**
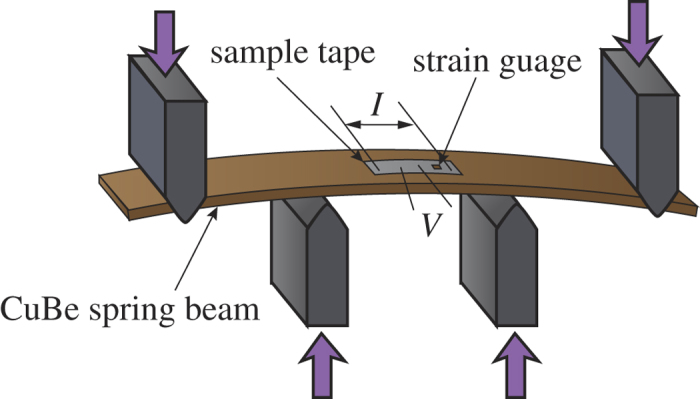
Schematic experimental set-up for the transport measurements under external strain using a four-point bending spring.

We measured the external-strain dependence of *T*_c_ for the detwinned RE123 tapes. The four-point bending spring method was used for the strain-dependence measurements^10^. A schematic experimental set-up is shown in Fig. 1. The sample tape strongly adhered to a Cu–Be spring beam with a 3-mm thickness, 66-mm length, and 10-mm width by a solder. The Cu–Be spring beam was placed into the four-point bending jig. Strain was applied by bending the Cu–Be spring beam. The applied strain of the sample tape was measured by a strain gauge, which was glued onto the sample. If the sample was mounted on the inside of the bending beam, a compressive strain can be applied. In the opposite case, the applied strain becomes tensile. Fig. 1 corresponds to the set-up for tensile strain. Because the thickness of the RE123 layer (≈ 1 μm) is much thinner than that of the spring beam (≈ 3 mm), the strain of the sample can be recognized as the axial strain. The resistivity of the sample was measured by a four-probe method with a current density of approximately 10 A/cm^2^. The current flows along the applied strain direction. The sample temperature was controlled by a He-gas-flow cryostat.

## Results and discussion

**Figure 2:**
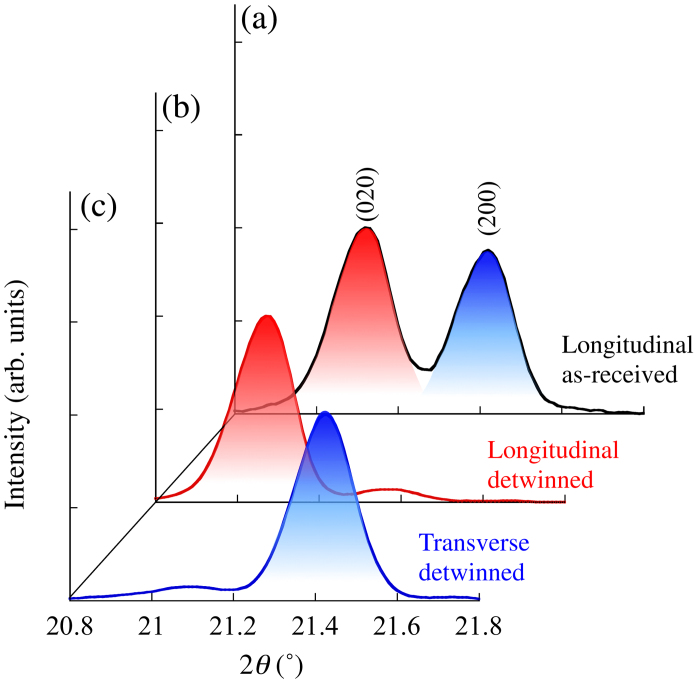
The (200) and (020) diffraction peaks before and after strain annealing (detwinning process): (**a**) longitudinal direction before strain annealing (as-received sample) and (**b**) longitudinal and (**c**) transverse directions after strain annealing (detwinned sample).

The X-ray diffraction patterns are shown in Fig. 2. In the ordinary-made (as-received) RE123 tape, the (020) and (200) peaks appear for the longitudinal direction, as shown in Fig. 2(a). This indicates that the *a* and *b* axes in the grains are aligned with the longitudinal direction of the tape owing to the twin boundaries. After strain annealing, however, the (200) peak disappears, and only the (020) peak remains for the longitudinal direction [Fig. 2(b)], whereas only the (200) peak remains without the (200) peak for the transverse direction [Fig. 2(c)]. Therefore, it is obvious that the *a* and *b* axes are completely aligned with the transverse and longitudinal directions, respectively. At the same time, the peak positions slightly shift after strain annealing in comparison with those of the as-received sample. This means that the residual strains also change because of strain annealing. The change in the residual strain is approximately 0.28% for the *b* axis in the longitudinal direction and –0.07% for the *a* axis in the transverse direction.

Unfortunately, many micro-cracks are observed along the transverse direction in the RE123 matrix and buffer layers, although no micro-cracks existed before the strain annealing. The density of the micro-cracks tends to depend on the applied strain at the annealing. When the applied strain for the annealing is smaller than 0.5%, the cracks were not induced. The micro-cracks align with the a axis of RE123. The size of a micro-crack in RE123 is typically 0.1 μm wide and 100–200 μm long. The density of micro-cracks in RE123 is smaller than that in the buffer layer. It is considered that the micro-cracks initially formed in the buffer layer and spread to the RE213 matrix. The critical current density *J*_c_ values at 77 K and self-field are approximately 0.79 MA/cm^2^ for the *a* axis and approximately 0.013 MA/cm^2^ for the *b* axis. These *J*_c_ values are more than one order of magnitude smaller than the value of approximately 2.6 MA/cm^2^ before strain annealing. The cracks may also influence the external-strain dependency of the superconducting properties along the *b* axis, which is perpendicular to the micro-cracks.

**Figure 3:**
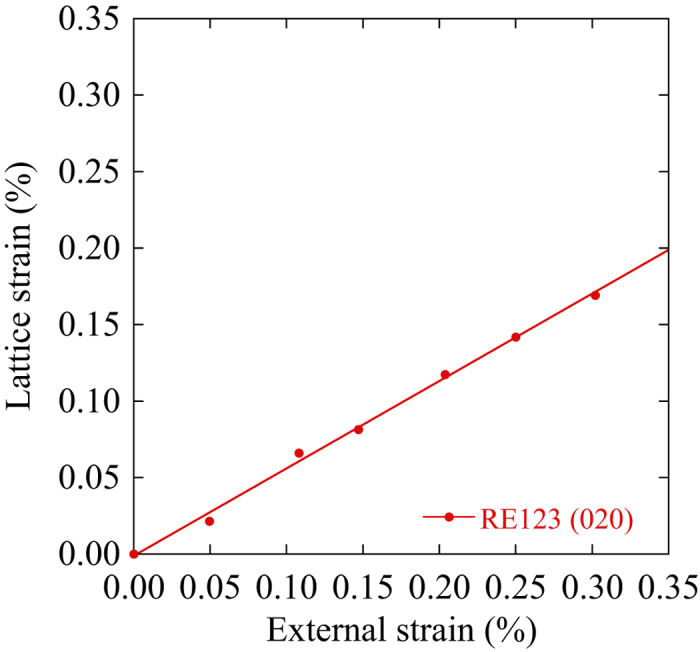
Relationship between the internal and external applied strains at room temperature. The slope of the solid line is 0.57.

Figure 3 shows the relationship between the internal and external strains for the *b* axis in the longitudinal direction. The external strain ε_ext_ was measured by the strain gauge on the sample tape surface. Hence, it is the same as the applied strain. The internal strains ε_int_* were determined by the change in the *d*-spacing for the (020) peak. In this case, an accuracy of the strain measurement is less than 0.01%. For the transverse direction, however, this experiment cannot be performed because the sample length is too short. The slope of the internal strain versus the macroscopic strain, *t* = ε_int_*/ε_ext_, was approximately 0.57 for the *b* axis. Therefore, approximately 57% of the applied strain is induced in the *b* axis of RE123. Even for the as-received RE123 tapes, *t* is not unity. It has been reported that the values of *t* are approximately 0.88 for the *a* axis and 0.9 for the *b* axis in the longitudinal direction^16^. According to Osamura *et al.*, the origin of these discrepancies is related to the micro-twin structure^17^. In this study, *t* ≈ 0.57 was obtained for the *b* axis experimentally, but we could not measure *t* for the *a* axis. The remaining 43% external strain may be induced in the micro-cracks, when the external strain is perpendicular to the crack directions. We assume *t* ≈ 1 for the *a* axis here because there are no twin boundaries.

**Figure 4:**
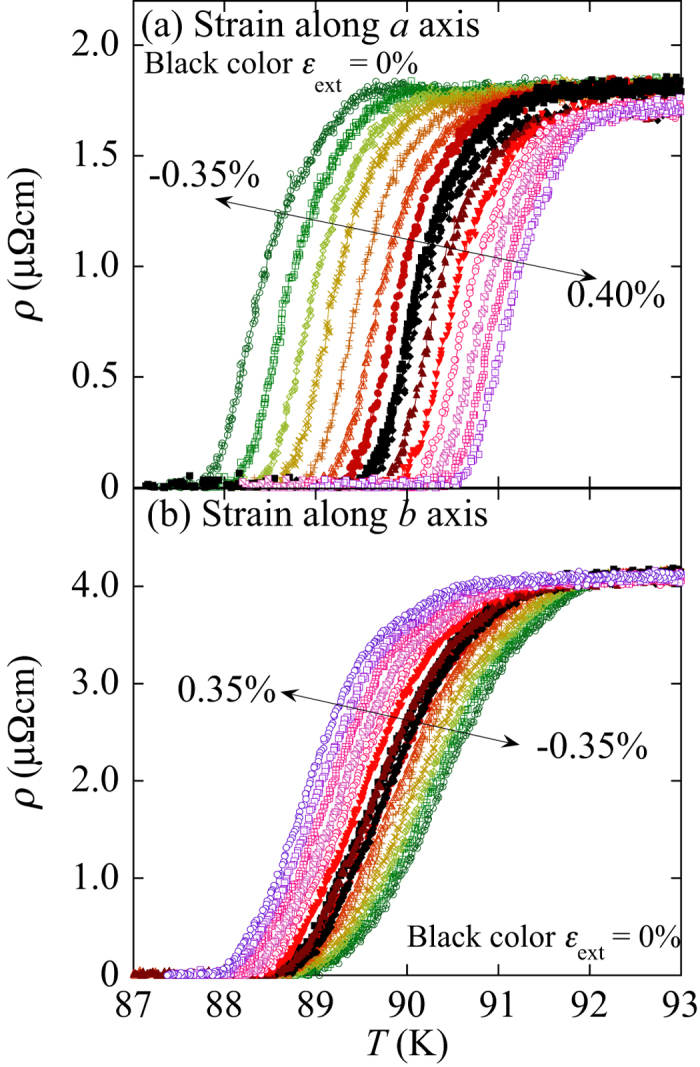
Temperature dependence of the resistivity at various strains along the (**a**) *a* axis and (**b**) *b* axis. The external applied strains are shown. The black symbols correspond to the zero strain data.

**Figure 5:**
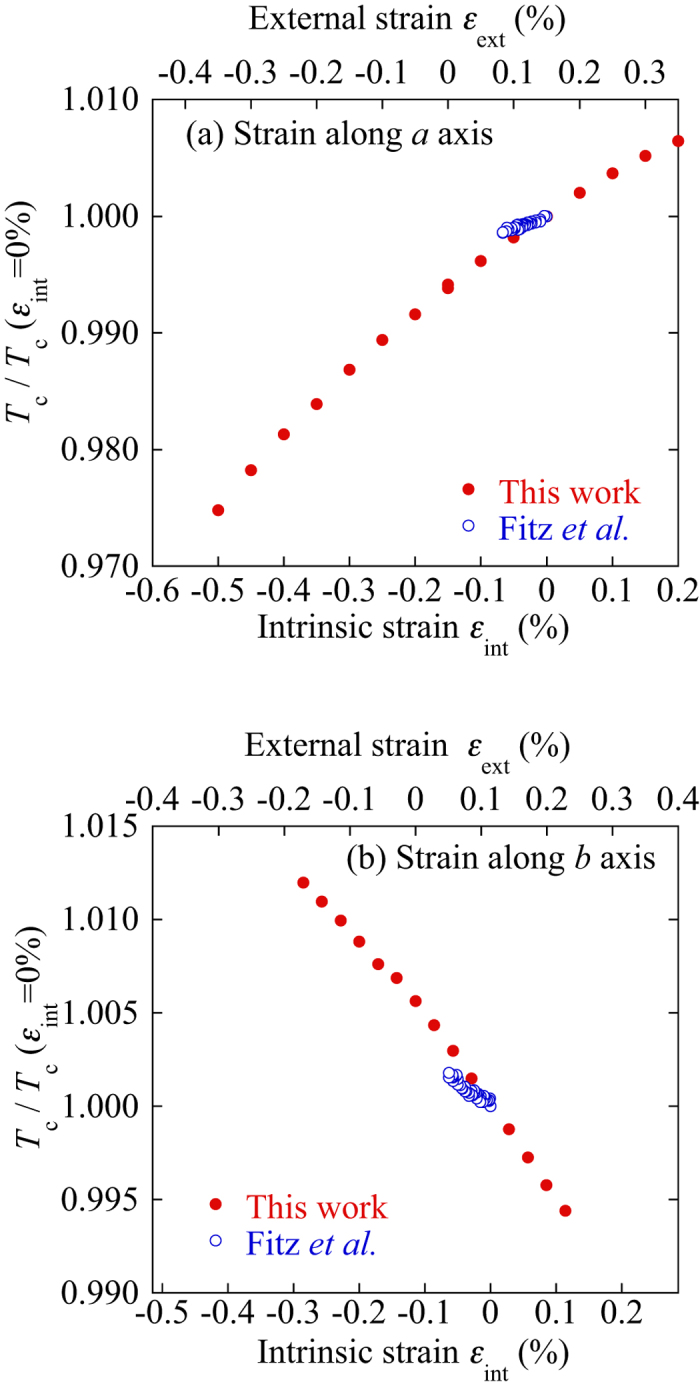
Critical temperature *T*_c_ normalized by the value at zero intrinsic strain for the applied strain along the (**a**) *a* axis and (**b**) *b* axis. Open circles indicate the single-crystal data estimated from the uniaxial-pressure dependence of *T*_c_ using the Young’s moduli of 162.7 GPa for the *a* axis and 178.1 GPa for the *b* axis.

Figure 4 shows the superconducting transitions of the resistivity at various applied strains. It’s to be noted that the normal resistivity of RE123 cannot be seen in this case because of existence of the silver cap layer. The decreases in the resistivity gradually shift depending on the applied strains. The superconducting transitions for the *b* axis are slightly broader than those for the *a* axis. This may be related to the micro-cracks perpendicular to the *b* axis. *T*_c_ was determined by the midpoint of the superconducting transition of the resistivity. The obtained *T*_c_ is plotted as a function of the strain in Fig. 5. The upper axis in the figure is the applied strain measured by the strain gauges on the sample. The thermal contraction difference between the RE123 tape and the Cu–Be plate, which was used as the bending spring for the strain measurements, is estimated to be approximately 0.15%. To calculate the intrinsic strain in RE123, the thermal contraction difference between the Cu–Be bending spring and RE123 should be taken into account in addition to *t*. The effect of the thermal contraction of the spring is estimated to be approximately 0.1% compression. Therefore, the intrinsic strain can be estimated by ε_int_ [%] = *t*(ε_ext_ – 0.15) = ε_int_*−0.15t. One should notice that the strain dependencies of *T*_c_ exhibit opposite slopes. This is similar to those for Y123 single crystals^3^. Because the opposite slopes for uniaxial strain dependences of *T*_*c*_ between *a* and *b* axes are also expected through the Poisson’s ratio, the effect of Poisson’s ratio should be taken into account. The Poisson’s ratio of Hastelloy, 0.3, was used here, since the thickness of the REBCO layer (0.001 μm) is much thinner than the Hastelloy (0.1 μm). If we consider the effects of transverse strains through the Poisson’s ratio, however, the strain dependences of *T*_*c*_ are different to those from another axes. It means that the opposite slopes of the uniaxial strain dependences of Tc indicate a physical meaning related to the superconducting mechanism. In order to compare the uniaxial-pressure dependence of *T*_c_ for the single crystal reported by Welp *et al.*, the strains are estimated from the uniaxial pressure using the Young’s moduli of 162.7 GPa for the *a* axis and 178.1 GPa for the *b* axis^9^. The strain dependencies of *T*_c_ for a single crystal are compared in Figs. 5(a,b). The slopes are almost similar to each other. However, the measured strain ranges in this study are 4–7 times larger than those reported in ref. 3. Therefore, we can conclude that the strain dependency of *T*_c_ obeys a power law instead of a linear relationship if we evaluate the strain effect for a wide enough strain region. The enhancement in *T*_c_ in RE123 can be obtained by compression for the *a* axis and tension for the *b* axis.

**Figure 6:**
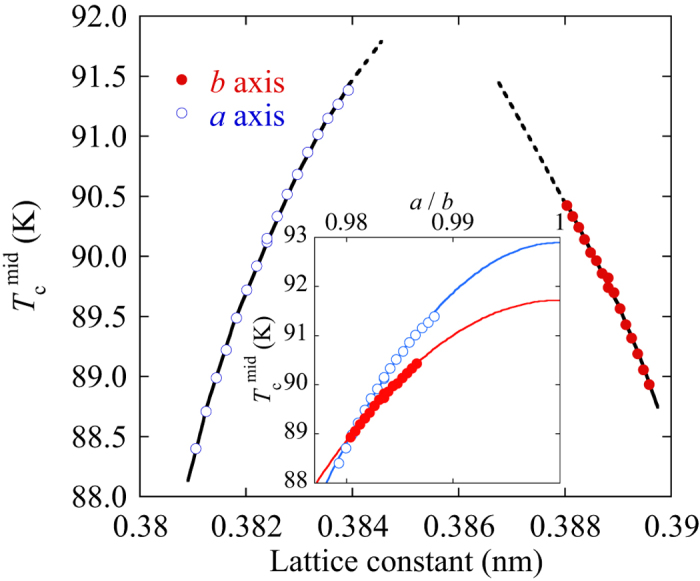
Relationship between *T*_c_ and the lattice constants at room temperature. The lines in the figure are guides for the eyes. The inset shows the *T*_c_ versus orthorhombicity *a/b*. The solid line in the inset is the result of fitting by a function of (*a/b*)^2^.

The *T*_c_ values are also plotted versus the lattice constants at room temperature in Fig. 6. The lattice constants were obtained at zero applied strain from the XRD data in Fig. 2 and calculated using the internal strain. Because the *b* axis is larger than the *a* axis, the lattice-constant dependence of *T*_c_ appears as a dome shape. A maximum *T*_c_ greater than 92 K is expected around a lattice constant of 0.385 nm. It is suggested that the tetragonal structure is preferable to obtain a higher *T*_c_. However, the variation of the other axes through Poisson’s ratio should be considered if we discuss the tetragonal nature. Because an RE123 layer is deposited onto the substrate, and the thickness of Hastelloy is much larger than those of the other layers, the Poisson’s ratio of 0.3 for Hastelloy has to be utilized to estimate the contribution of other crystal axes. Then, the ratio of the lattice constants between *a* and *b* axes can be estimated from the internal strains. The inset of Fig. 6 shows the relationship between *T*_c_ and the value of *a/b*, which corresponds to the orthorhombicity of RE123 crystals. Both the *a*- and *b*-axis strain dependences of *T*_c_ seem to attain a maximum at *a/b* ≈ 1, i.e. a tetragonal structure. Therefore, it is considered that the square CuO_2_ plane in the RE123 lattice with *a* = *b* ≈ 0.385 nm is the optimum conditions for high *T*_c_. The maximum *T*_c_ values at *a*/*b* = 1 in the *a-*axis is slightly higher than that in the *b*-axis. It may be due to the difference of lattice parameters between the *a*- and *b*-axes at *a*/*b* = 1. The five basic requirements for enhancing *T*_c_ have been proposed previously^4^,^18^. According to MacManus-Driscoll and Wimbush, ‘Make the CuO_2_ planes flat, square, and of optimal size’ is one of the requirements. The obtained data in the inset of Fig. 6 strongly suggest that the square of the CuO_2_ plane is the best for realizing a high *T*_c_. In addition, it has been suggested that the optimal in-plane lattice parameter is approximately 0.384–0.385 nm for a high *T*_c_^18^. Our obtained result is very close to the optimum lattice parameter. In this case, however, the distance between the CuO_2_ plane and apical oxygen and the hole concentration are not clear yet. If we can optimize these parameters, *T*_c_ of RE123 would be further enhanced. The mechanism of the anisotropy in the uniaxial strain effects on *T*_c_ along the *a* and *b* axes is not clear yet. One candidate is the model based on the van Hove singularly (VHS)^19^. According to Chen *et al.*, the maximum *T*_c_ possibly reflects the change in the VHS around (π, 0). When the lattice constants along the *a* and *b* axes are equal, the VHS at (π, 0) is strongest in *d*-wave superconductivity. The results obtained here are in good agreement with the VHS model.

In summary, we determined the uniaxial-strain dependence along the *a* and *b* axes in RE123-coated conductors over a wide range of strain from compression to tension. We found that the strain dependencies for the *a* and *b* axes obey a power-law but exhibit opposite slopes. In particular, it has been proven experimentally that the optimum conditions of the CuO_2_ plane in RE123 are a square with a lattice constant of 0.385 nm to attain a high critical temperature.

## Additional Information

**How to cite this article**: Awaji, S. *et al.* Strain-controlled critical temperature in REBa_2_Cu_3_O_y_-coated conductors. *Sci. Rep.* **5**, 11156; doi: 10.1038/srep11156 (2015).
